# Identification of Long Intergenic Noncoding RNAs in *Rhizoctonia cerealis* following Inoculation of Wheat

**DOI:** 10.1128/spectrum.03449-22

**Published:** 2023-04-10

**Authors:** Ke Yi, Weiyi Yan, Xiang Li, Shuqing Yang, Jiaqi Li, Yifan Yin, Fengping Yuan, Haiying Wang, Zhensheng Kang, Dejun Han, Qingdong Zeng

**Affiliations:** a State Key Laboratory of Crop Stress Biology for Arid Areas, Northwest A&F University, Yangling, Shaanxi, China; Beijing Forestry University

**Keywords:** wheat sharp eyespot, lincRNAs, virulence, disease control, HIGS

## Abstract

Wheat sharp eyespot caused by Rhizoctonia cerealis is primarily a severe threat to worldwide wheat production. Currently, there are no resistant wheat cultivars, and the use of fungicides is the primary method for controlling this disease. Elucidating the mechanisms of R. cerealis pathogenicity can accelerate the pace of the control of this disease. Long intergenic noncoding RNAs (lincRNAs) that function in plant-pathogen interactions might provide a new perspective. We systematically analyzed lincRNAs and identified a total of 1,319 lincRNAs in *R. cerealis*. We found that lincRNAs are involved in various biological processes, as shown by differential expression analysis and weighted correlation network analysis (WGCNA). Next, one of nine hub lincRNAs in the blue module that was related to infection and growth processes, *MSTRG.4380.1*, was verified to reduce *R. cerealis* virulence on wheat by a host-induced gene silencing (HIGS) assay. Following that, RNA sequencing (RNA-Seq) analysis revealed that the significantly downregulated genes in the *MSTRG.4380.1* knockdown lines were associated mainly with infection-related processes, including hydrolase, transmembrane transporter, and energy metabolism activities. Additionally, 23 novel microRNAs (miRNAs) were discovered during small RNA (sRNA) sequencing (sRNA-Seq) analysis of *MSTRG.4380.1* knockdown, and target prediction of miRNAs suggested that *MSTRG.4380.1* does not act as a competitive endogenous RNA (ceRNA). This study performed the first genome-wide identification of *R. cerealis* lincRNAs and miRNAs. It confirmed the involvement of a lincRNA in the infection process, providing new insights into the mechanism of *R. cerealis* infection and offering a new approach for protecting wheat from *R. cerealis*.

**IMPORTANCE** *Rhizoctonia cerealis*, the primary causal agent of wheat sharp eyespot, has caused significant losses in worldwide wheat production. Since no resistant wheat cultivars exist, chemical control is the primary method. However, this approach is environmentally unfriendly and costly. RNA interference (RNAi)-mediated pathogenicity gene silencing has been proven to reduce the growth of *Rhizoctonia* and provides a new perspective for disease control. Recent studies have shown that lincRNAs are involved in various biological processes across species, such as biotic and abiotic stresses. Therefore, verifying the function of lincRNAs in *R. cerealis* is beneficial for understanding the infection mechanism. In this study, we reveal that lincRNAs could contribute to the virulence of *R. cerealis*, which provides new insights into controlling this pathogen.

## INTRODUCTION

The necrotrophic fungus Rhizoctonia cerealis, belonging to the binucleate *Rhizoctonia* subgroup AG-DI, is the primary causal agent of wheat sharp eyespot. In addition, it can cause many other diseases (1–3). When infecting wheat plants, R. cerealis survives in the soil and damages the transport tissues in stems and sheaths (4). *R. cerealis* has been considered a critical worldwide pathogen affecting wheat crops for a few decades and is a significant threat to wheat production (5). For example, in China, wheat sharp eyespot caused more than 15 million dollars in economic damage from 2005 to 2008 (4), and in the decade since 2005, this disease affected 9 million hectares of wheat (6). In addition, wheat sharp eyespot disease has resulted in field losses in other countries such as Chile (7).

A reliable approach for controlling this disease is breeding resistant varieties (4). However, there are still no viable resistant cultivars for this disease (5, 6) despite many efforts to study resistance to *R. cerealis* at the wheat level. The use of fungicides is still the primary method for controlling this disease (5, 8), negatively affecting the environment and increasing the cost to farmers. Revealing the molecular basis of *R. cerealis* development and infection may provide new insights into the control of this disease. Long noncoding RNAs (lncRNAs), noncoding RNAs (ncRNAs) with long transcripts (>200 nucleotides [nt]) and limited protein-coding potential (9–11), have served as essential regulators of transcription in various biological processes, including growth and development responses to abiotic and biotic stresses, with many studies on animals and plants (12, 13). Based on genome location, lncRNAs are divided into three categories, long intergenic noncoding RNAs (lincRNAs), intronic noncoding RNAs (incRNAs), and long noncoding natural antisense transcripts (lncNATs) (14). Several recent studies have demonstrated that lncRNAs play essential roles in plant-pathogen interactions, most of which are lncRNAs in the host (15–20). In contrast, there are just a few studies on lncRNAs in pathogens. An lncNAT, *UvlncNATMFS*, has been shown to regulate growth and stress responses in the phytopathogen Ustilaginoidea virens (13). Functional verification of *lncRNA009491* and *lncRNA012077* suggests that lncRNAs regulate virulence in Verticillium dahlia through positive and negative regulation (21). The roles of lncRNAs in phytopathogens could be used to alleviate plant diseases caused by pathogens. However, few lncRNAs have been functionally validated in fungi, mainly in yeasts (22, 23). Therefore, exploring the roles of lncRNAs in *R. cerealis* may provide a new perspective to control the disease caused by *R. cerealis* and can also provide some information for better studying their roles in pathogenic filamentous fungi.

At present, there has been no report on lncRNAs in *R. cerealis*, and it is unknown whether lncRNAs play a role in *R. cerealis* development and infection. To address this question, we report the genome-wide identification of *R. cerealis* lincRNAs to identify lincRNAs potentially essential for development and infection processes. We predicted the functions of genes coexpressed with lincRNAs and identified a lincRNA that could affect the virulence of *R. cerealis*. Finally, we revealed that the lincRNA could regulate genes involved infection-related processes, not as a competitive endogenous RNA (ceRNA). Our findings could help elucidate how lincRNAs regulate *R. cerealis* infection of wheat and provide clues for disease control.

## RESULTS

### Identification, characterization, and expression profiles of lincRNAs in *R. cerealis*.

**FIG 1:**
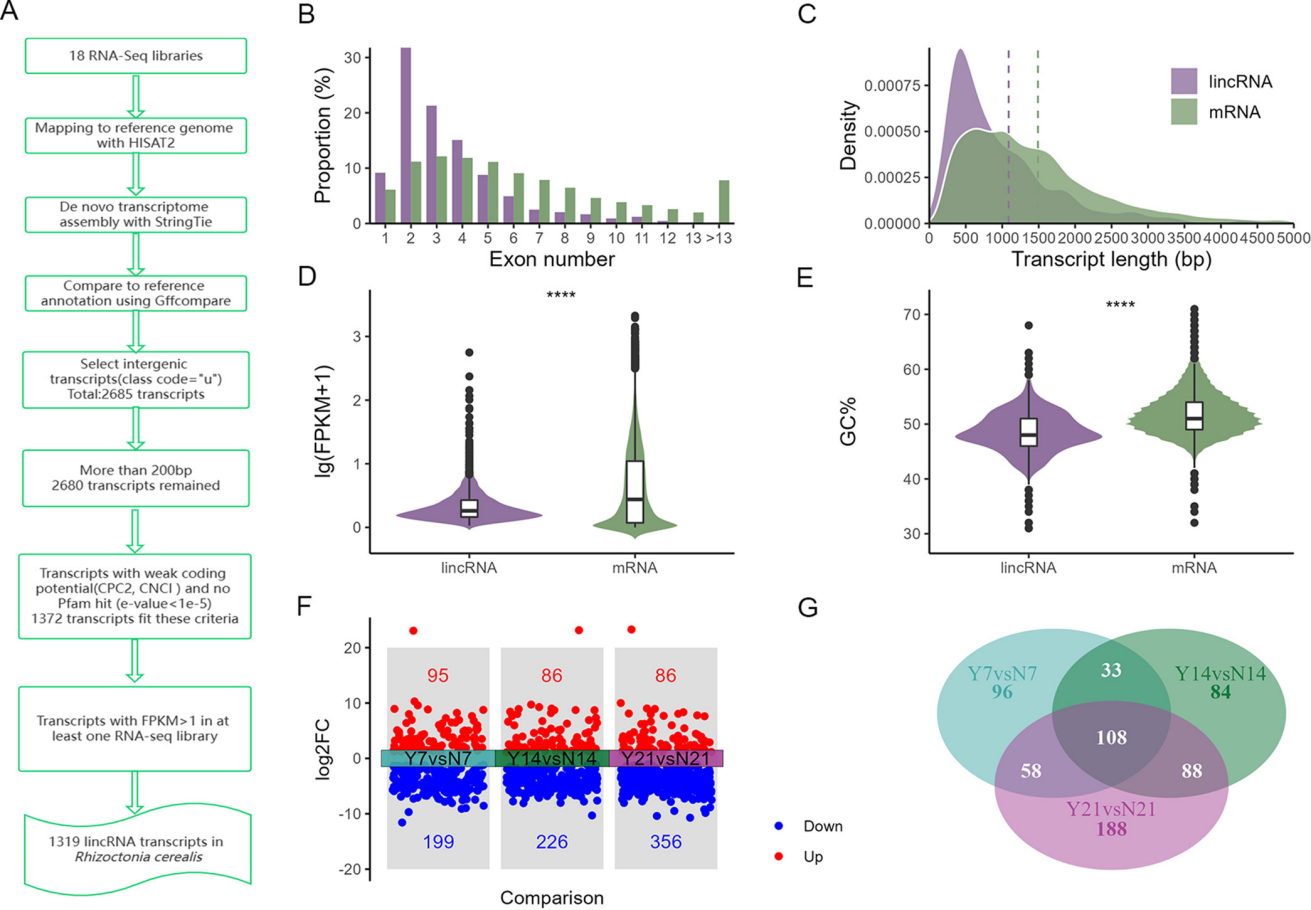
Identification, characterization, and expression profiles of lincRNAs in *R. cerealis*. (A) Pipeline for the identification of lincRNAs in *R. cerealis*. The details of each step are described in Materials and Methods. (B) Distribution of the numbers of exons in mRNAs and lincRNAs. (C) Distribution of lengths of mRNAs and lincRNAs. Purple and green dashed lines represent the average lengths of lincRNAs and mRNAs, respectively. (D and E) Expression levels (D) and GC contents (E) of mRNAs and lincRNAs. ****, *P* < 0.0001 (the *P* value was calculated using a Wilcox test). (F) Up- and downregulated lincRNAs in the treatment group versus the control group. log2FC, log_2_ fold change. (G) Venn diagram showing the overlap and nonoverlap of differentially expressed lincRNAs from three comparisons. Y7, Y14, and Y21 refer to the treatments of *R. cerealis*-infected Yangmai 58 wheat for 7 days, 14 days, and 21 days, respectively. N7, N14, and N21 refer to the controls where *R. cerealis* was cultured on medium for 7 days, 14 days, and 21 days, respectively.

To identify lincRNAs confidently in the *R. cerealis* genome, we performed the following transcript-filtering steps (Fig. 1A). First, transcripts of >200 nt long with class code “u” were chosen after annotating the assembled transcripts using Gffcompare, and a total of 2,680 transcripts were retained. Next, we kept 1,372 transcripts categorized as “noncoding” based on CPC2 (Coding Potential Calculator 2) and CNCI (Coding-Noncoding Index) and with no Pfam hit by Pfamscan (see Fig. S1 in the supplemental material). Subsequently, we removed 53 low-confidence lincRNAs (fragments per kilobase per million [FPKM] value of <1 in all RNA sequencing [RNA-Seq] libraries). Finally, a total of 1,319 transcripts were identified as lincRNAs in *R. cerealis* (Data Set S1).

To characterize the genomic features of the lincRNAs in *R. cerealis*, we analyzed the exon distributions, lengths of the transcripts, gene expression levels, and GC contents of these lincRNAs and mRNAs. lincRNAs had an average of 3.7 exons, significantly fewer than the 6.4 exons found in mRNAs. Only 41.3% of the mRNAs had fewer than 4 exons, whereas 77.3% of the lincRNAs had fewer than 4 exons (Fig. 1B). Furthermore, two-exon lincRNA transcripts were the most abundant in this study. The mean and median lengths of lincRNAs were 1,132 bp and 783 bp, respectively, shorter than those of mRNAs, 1,490 bp and 1,238 bp, respectively. Overall, the lincRNA size distribution ranged from 200 to 7,842 bp, with 62% ranging from 201 to 1,000 bp, while most mRNAs (about 61.3%) were more than 1,000 bp long (Fig. 1C). In terms of FPKM values, the expression level of lincRNAs (mean, 2.8) was notably lower than that of mRNAs (mean, 15.89) (Fig. 1D). In addition, the GC content in lincRNAs (48.6%) was significantly lower than that in mRNAs (52%) (Fig. 1E). To determine whether *R. cerealis* lincRNAs could contribute to the infection process, we examined the expression of lincRNAs after inoculation (treatment) or noninoculation (control) of wheat at 7, 14, and 21 days postinoculation (dpi), respectively. We discovered that 654 lincRNAs were differentially expressed in the treatment group compared to the control group (false discovery rate [FDR] of <0.05 and absolute log_2_ fold change [|log_2_ fold change|] of ≥1). Of these 654 differentially expressed lincRNAs (DELs), 294, 312, and 442 lincRNAs were differentially expressed at 7, 14, and 21 dpi, respectively (Fig. 1F). Most of these DELs were downregulated, and the number of differentially expressed lincRNAs increased with increasing durations of infection. Totals of 96, 84, and 188 DELs were unique and differentially expressed at 7 dpi, 14 dpi, and 21 dpi, respectively (Fig. 1G). Especially at 21 dpi, about 42.5% of the DELs (442) were unique. One hundred eight of these DELs were shared by all three infection stages. These findings indicated that *R. cerealis* lincRNAs might play essential roles in infection.

### Functional analysis of infection and development associated with lincRNAs.

**FIG 2:**
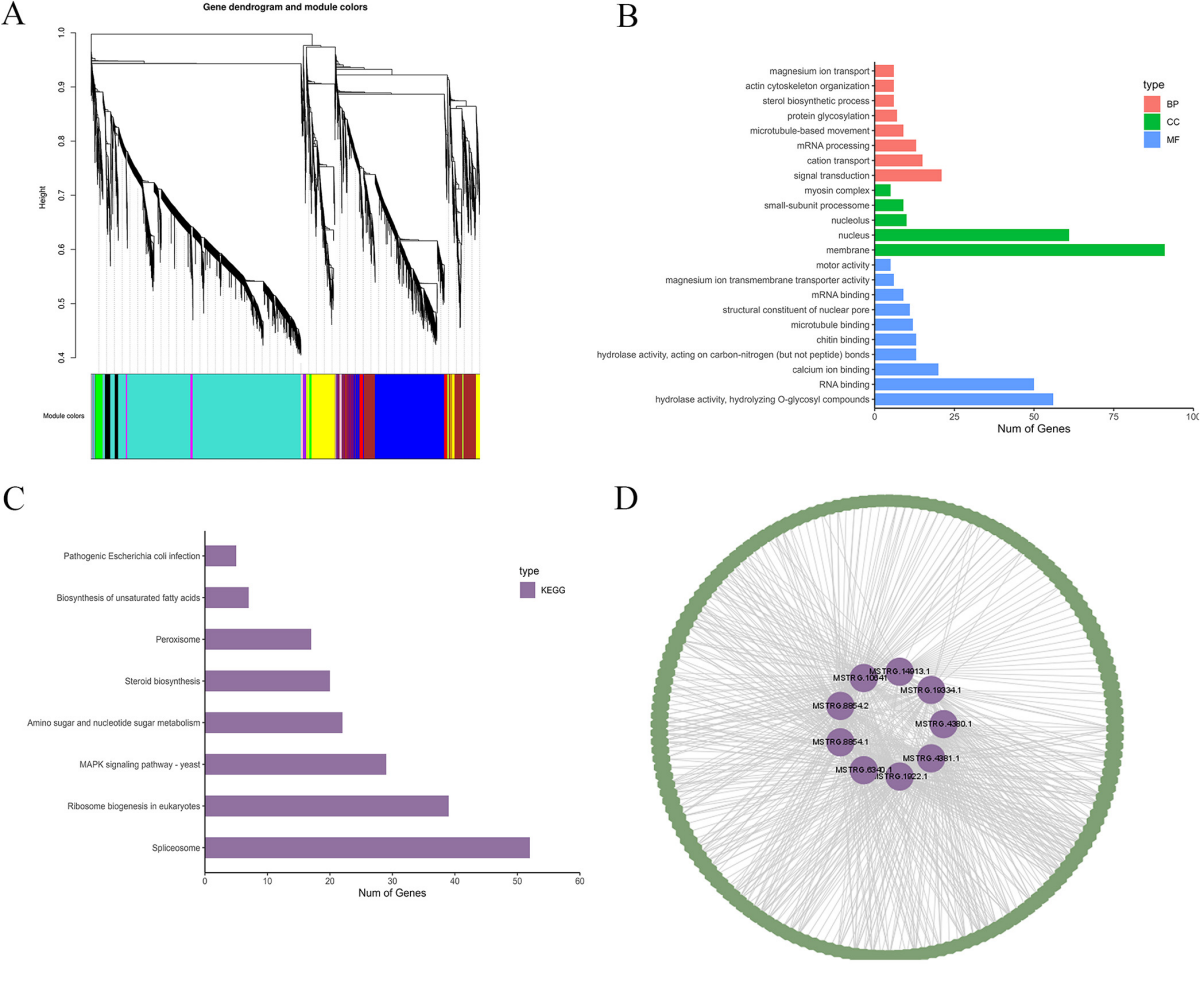
Identification of hub lincRNAs involved in the processes of infection and growth. (A) Clustering dendrogram of differentially expressed lincRNAs and differentially expressed genes. Different colors refer to the different modules described in Table 1. (B) Biological process (BP), cellular component (CC), and molecular function (MF) items enriched in the blue module. (C) KEGG pathway analysis of the genes enriched in the blue module. (D) Nine hub lincRNAs with high intramodular connectivity were selected and visualized by using Cytoscape. Purple represents lincRNA nodes; green represents gene nodes.

To predict the putative function of lincRNAs in the infection process of *R. cerealis*, a coexpression network was constructed to associate 654 DELs and 10,520 differentially expressed genes (DEGs) derived from a previous study by Zeng et al. (24). A total of 11 modules (except for the gray module), which captured 624 DELs and 10,439 DEGs, were identified (Table 1). To verify whether lincRNAs are involved in the development- and infection-related processes of *R. cerealis*, the Gene Ontology (GO) and KEGG pathway databases were used to analyze the genes coexpressed with lincRNAs in each module (Data Set S2). Among these 11 modules, genes in the blue module were significantly enriched in the steroid biosynthesis (GO:0016126), biological process and steroid biosynthesis (Ko00100), mitogen-activated protein kinase (MAPK) signaling (Ko04011), and pathogenic Escherichia coli infection (Ko05130) pathways (Fig. 2B and C). These findings suggested that the lincRNAs in the blue module likely regulated *R. cerealis* growth and infection. To further screen potential key lincRNAs in this module, we finally identified nine hub lincRNAs in the blue module with the criteria of an intramodular gene connectivity value of >0.4 and a degree of >50 (Fig. 2D).

**TABLE 1:** Numbers of lincRNAs and mRNAs in 11 modules^*a*^

Module^*a*^	No. of lincRNAs	No. of mRNAs	Total no. of RNAs
Black	19	244	263
Blue	65	2,209	2,274
Turquoise	399	4,961	5,360
Red	18	258	276
Brown	46	1,167	1,213
Green	8	283	291
Green-yellow	8	76	84
Magenta	5	116	121
Pink	2	131	133
Purple	11	78	89
Yellow	43	916	959

Total	624	10,439	11,063

a Different colors represent different coexpression modules from the results of WGCNA.

### Confirmation of the expression patterns of hub lincRNAs.

**FIG 3:**
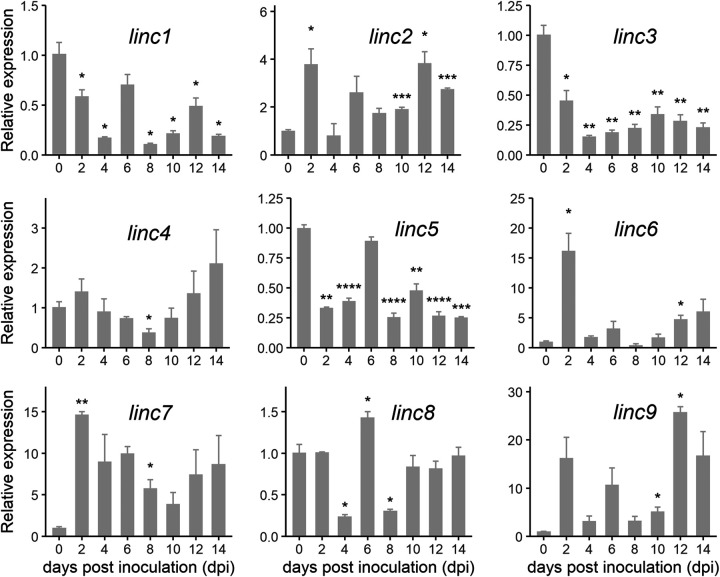
Relative expression levels of the nine hub lincRNAs following *R. cerealis* infection. The hyphae of *R. cerealis* cultured on PDA were used as a control. Relative expression levels were calculated by the 2^−ΔΔ^*^CT^* method. Data were normalized to the transcript level of *RcActin*, with hyphae of *R. cerealis* set to a value of 1. The values represent the means ± standard errors (SE) (*n* = 3) from three biological replicates. *linc1* to *linc9* represent *MSTRG.1922.1*, *MSTRG.4381.1*, *MSTRG.6340.1*, *MSTRG.8854.1*, *MSTRG.8854.2*, *MSTRG.10641.2*, *MSTRG.19334.1*, *MSTRG.14913.1*, and *MSTRG.4380.1*, respectively.

We used quantitative real-time PCR (qRT-PCR) to examine the temporal expression patterns of these nine lincRNAs at 0, 2, 4, 6, 8, 10, 12, and 14 dpi (Fig. 3). Compared to the *RcActin* gene, *linc2*, *linc6*, *linc7*, and *linc9* were significantly upregulated. At the same time, *linc1*, *linc3*, and *linc5* were significantly downregulated. Generally, disease-related genes are highly expressed in the early stage of infection. Additionally, at 2 dpi, the *linc6*, *linc7*, and *linc9* expression levels were 16, 23, and 16 times higher than those of *RcActin*, suggesting that these three lincRNAs may be essential for the process of *R. cerealis* infection. Therefore, these three lincRNAs were selected for further analysis.

### *MSTRG.4380.1* conferred pathogenicity to wheat.

**FIG 4:**
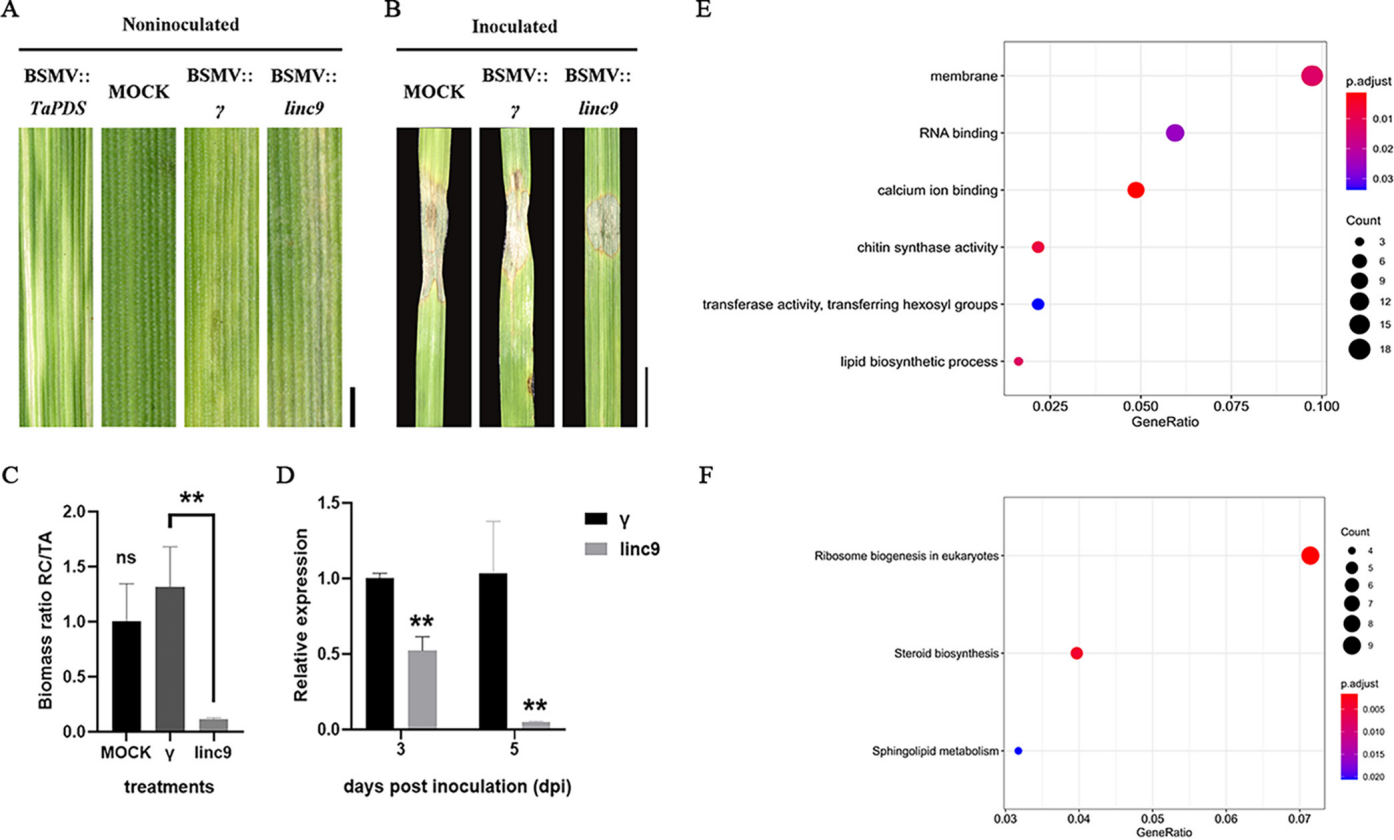
Functional verification of *MSTRG.4380.1* and prediction of regulatory processes in which *MSTRG.4380.1* is involved. (A) Mild chlorotic mosaic symptoms at 14 dpi were observed on the fourth leaves of wheat inoculated with BSMV. Wheat leaves infected with BSMV::*TaPDS*, which were used as the positive control, showed a photobleaching phenotype. Mock-inoculated plants showed no change in phenotype on the fourth leaves. Bars, 0.5 cm. (B) Disease phenotypes of the fourth leaves inoculated with *R. cerealis*. BSMV::γ- and mock-inoculated plants were the negative controls. Bars, 2 cm. (C) The fungal/wheat biomass ratio was determined by measuring the total DNA content of inoculated wheat leaves at 7 dpi using the absolute quantification of the internal reference genes *RcActin* (RC) and *TaEF* (TA). The values represent the means ± SE (*n* = 3) from three biological replicates. ns, not significant. (D) Relative expression levels of *linc9* and *MSTRG.4380.1*. Data were normalized to the transcript level of *RcActin*, with the expression level of BSMV::γ set to a value of 1. **, *P* < 0.01 (the *P* value was calculated using Student’s *t* test). (E and F) GO (E) and KEGG pathway (F) analyses of genes that were coexpressed with *MSTRG.4380.1* and met the criterion of an intramodular gene connectivity value of >0.3.

Due to the severe lag in functional genomics studies, there is no transformation system for *R. cerealis*. To further demonstrate whether these three hub lincRNAs were involved in the infection process, we knocked down their expression using the barley stripe mosaic virus host-induced gene silencing (BSMV-HIGS) system, which is widely used to verify the function of pathogenicity genes in fungi. At 14 dpi, the BSMV-infected wheat showed mild chlorotic mosaic symptoms (Fig. 4A), and the endogenous phytoene desaturase (PDS) gene was used as the positive control for BSMV infection symptoms. Subsequently, the leaves showing mild chlorotic mosaic symptoms were selected for *R. cerealis* infection. According to statistical analysis, only the lesion areas on the leaves of the inoculated *linc9* HIGS plants showed macroscopic reductions compared to the negative controls (Fig. 4B). In contrast, the lesion areas of the plants with silenced *linc6* and *linc7* did not show a significant difference compared to the control. The biomass of *R. cerealis* decreased significantly in *linc9*-silenced plants (Fig. 4C). Furthermore, qRT-PCR analysis revealed that endogenous lincRNA was downregulated considerably in BSMV::*linc9* HIGS plants (Fig. 4D). These results suggest that the silencing of *linc9* could dramatically reduce the pathogenicity of *R. cerealis*, and this was selected for intensive study. Moreover, to elucidate the specific process that *MSTRG.4380.1* participates in, we retrieved its coexpressed genes with the criterion of an intramodular gene connectivity value of >0.3. GO analysis indicated that these genes were enriched in categories related to membrane (GO:0016020), RNA binding (GO:0003723), calcium ion binding (GO:0005509), chitin synthase activity (GO:0004100), transferase activity, transferring hexosyl groups (GO:0016758), and lipid biosynthetic process (GO:0008610). KEGG pathway analysis showed that these genes were involved in the ribosome biogenesis (Ko03008), steroid biosynthesis (Ko00100), and sphingolipid metabolism (Ko00600) pathways (Fig. 4E and F). Because lncRNA preferentially regulates the expression of nearby genes, we chose coding genes 10 kb upstream and 20 kb downstream of *MSTRG.4380.1* as its *cis* target genes (Data Set S3A). Next, we intersected these genes with their coexpressed genes (Data Set S3B), but no candidate genes were retained, indicating that *MSTRG.4380.1* regulates genes in *trans*.

### RNA-Seq analysis of *linc9* knockdown.

**FIG 5:**
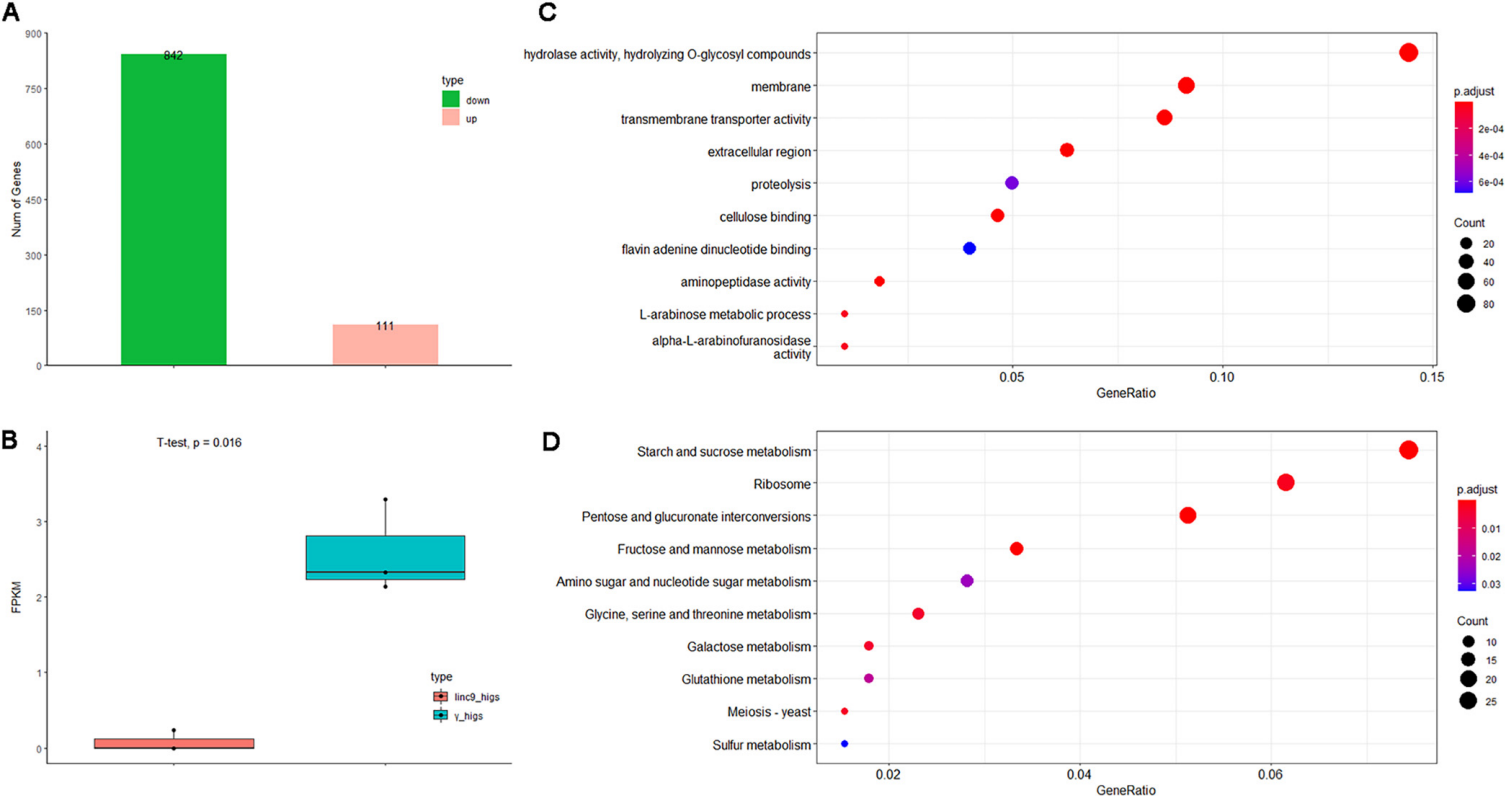
*MSTRG.4380.1* positively regulates *R. cerealis* virulence in wheat. (A) Numbers of significantly differentially expressed up- and downregulated genes between BSMV::*linc9* and BSMV::γ plants. (B) The transcript expression of *linc9* was silenced by HIGS compared with the control. (C and D) GO (E) and KEGG pathway (F) analyses of downregulated genes.

Moreover, to identify genes regulated by *linc9*, we conducted RNA-Seq analysis with infected wheat leaves collected at 5 dpi when the expression of *linc9* was significantly knocked down compared with the control (Fig. 4D). A total of 953 differently expressed genes were identified in *R. cerealis* (|log_2_ fold change| of ≥1 and adjusted *P* [*P*_adj_] value of ≤0.05), including 111 upregulated and 842 downregulated genes (Fig. 5A). Compared with the wheat inoculated with BSMV::γ, the expression of *linc9* was significantly reduced in BSMV::*linc9* plants (Fig. 5B). These results further demonstrated that HIGS could knock down the expression of *linc9* and also indicated that the knockdown of *linc9* could affect gene expression in *R. cerealis*. To determine the processes that *linc9* is involved in, we implemented GO and KEGG pathway enrichment analyses with 842 downregulated and 111 upregulated genes. Interestingly, GO enrichment analysis of 842 downregulated genes showed that these genes were located mainly in the membrane and extracellular region and were associated with infection-related processes, including hydrolase activity and transmembrane transporter activity (Fig. 5C). KEGG enrichment analysis showed that these genes were associated with energy metabolism, such as starch and sucrose metabolism and amino sugar and nucleotide sugar metabolism (Fig. 5D). However, GO enrichment analysis showed that these upregulated genes were enriched in the glycolytic process and proton transmembrane transporter categories, and no KEGG item was enriched for these genes (Fig. S2). These results indicate that *linc9* may positively regulate *R. cerealis* infection by regulating genes involved in hydrolase, transmembrane transporter, and energy metabolism activities.

### Identification and target prediction of miRNAs.

lncRNAs can act as ceRNAs competing with target transcripts for microRNAs (miRNAs), which interferes with the function of miRNAs (25). To verify whether *linc9* functions as a ceRNA, we conducted a small RNA (sRNA) sequencing (sRNA-Seq) analysis with infected wheat leaves collected at 5 dpi when the expression of *linc9* was significantly knocked down compared with the control. After quality control, a total of 227,414,158 clean reads were retained, for which the length distribution of reads in each sample is shown in Fig. S3. After filtering rRNA, tRNA, snRNA, and snoRNA, the retained reads were used to identify novel miRNAs by miRDeep2, resulting in a total of 23 miRNAs being identified (Data Set S4). Next, we verified whether these miRNAs could target *linc9* by using psRobot (26) and found that no miRNAs identified here targeted *linc9*, which indicates that *linc9* does not regulate *R. cerealis* infection as a ceRNA.

## DISCUSSION

*R. cerealis* is a devastating pathogen threatening worldwide wheat production (5, 8). Although some progress has been made on the mechanism of resistance of wheat to *R. cerealis* (6), there are no wheat cultivars resistant to *R. cerealis* at the moment. Currently, the use of fungicides is the primary measure for the control of this disease, although fungicide use can cause environmental pollution and increase labor costs. Revealing the mechanism of *R. cerealis* infection and development may provide clues for disease control. lncRNAs play critical roles in diverse biological processes in fungi, and lncRNAs may help gain new insights into the pathogen infection mechanism. Here, we performed genome-wide identification and functional prediction of *R. cerealis* lincRNAs during infection stages and discovered a lincRNA, *MSTRG.4380.1*, that reduced pathogenicity after its expression was silenced by the regulation of genes associated with infection-related processes.

lncRNAs have been shown to play critical roles in diverse biological processes in a wide range of species. However, as for plant-pathogenic filamentous fungi, there are only a few studies on lncRNAs, including those of Ustilago maydis (27), Fusarium graminearum (28), *Ustilaginoidea virens* (13), *Verticillium dahlia* (21), and Magnaporthe oryzae (29, 30). Naturally, the roles that lncRNAs can play in filamentous pathogenic fungi are less well known. Here, we performed genome-wide identification of *R. cerealis* lincRNAs, identifying a total of 1,319 lincRNAs. In addition, we found that *R. cerealis* lincRNAs may be involved extensively in multiple biological processes, for which half of the lincRNAs were differentially expressed during the different infection stages. These findings will provide the basis for further research on the role of *R. cerealis* lincRNAs and a reference for research on other pathogens.

It is challenging to confirm the role of lncRNA in RNA-mediated gene regulation (31). Although thousands of lncRNAs have been identified in phytopathogens (13, 21, 29, 32–34), only a few lncRNAs have been functionally characterized (13, 21, 33). Besides, functional genomics studies have lagged seriously, resulting from the lack of a genetic transformation system, which has hindered the functional verification of *R. cerealis* genes. Since the discovery of transkingdom RNA silencing, however, HIGS has been exploited as a powerful genetic tool to validate the function of pathogenicity genes during infections and has been applied to a variety of pathogenic fungi, including Blumeria graminis (35), Puccinia triticina (36), Fusarium graminearum (37), Puccinia striiformis f. sp. *tritici* (38), *Verticillium dahlia* (39), and Phakopsora pachyrhizi (40). Our latest work confirmed that HIGS can be applied to *R. cerealis* as a functional characterization tool (41). Here, A lincRNA, *MSTRG.4380.1*, was verified to affect the virulence of *R. cerealis* by HIGS, which reduced pathogenicity after its expression was silenced. Spray-induced gene silencing (SIGS), which was also confirmed to be a practical tool for research on *R. cerealis* virulence genes (41), has recently served as an innovative method of crop protection (42–44). By combining HIGS and SIGS technologies, this discovery may provide new insight into the control of wheat sharp eyespot.

Generally, lncRNAs may regulate the expression of neighboring genes in *cis* and distant genes in *trans* (45, 46), while most of the lncNATs regulate neighboring genes in *cis*. For example, *UvlncNATMFS* might affect the growth of *Ustilaginoidea virens* by regulating its associated sense gene *UvMFS* (13). In contrast to lncNATs, the expression of lincRNAs is not significantly correlated with that of their neighboring genes (47), which is further supported by our results. The coexpressed coding genes of *MSTRG.4380.1* were enriched in the membrane and involved in chitin synthase activity and steroid biosynthesis, indicating that these potential targets might contribute to infection-related processes and membrane formation. After knocking down *MSTRG.4380.1*, the downregulated genes were enriched mainly in hydrolase activity, transmembrane transporter activity, and energy metabolism activities, which further supports that *MSTRG.4380.1* regulates genes involved in infection-related processes. Additionally, lncRNAs can also serve as ceRNAs to interfere with miRNA function by competing with target genes for miRNAs (25). However, in this study, our results suggested that for *MSTRG.4380.1*, this was not the case. Nonetheless, the 23 novel miRNAs identified in *R. cerealis* may provide a basis for the analysis of transkingdom miRNAs in the *R. cerealis*-wheat interaction.

In conclusion, this first identification of lincRNAs and miRNAs in *R. cerealis* will provide a basis for studying the roles of lincRNAs and miRNAs in *R. cerealis*-wheat interactions. It was verified that the lincRNA *MSTRG.4380.1* could reduce the virulence of *R. cerealis* by regulating genes involved in infection-related processes. This may provide new insight into the control of wheat sharp eyespot by combining HIGS and SIGS technologies. However, much effort is still needed to understand the specific regulatory mechanism of *MSTRG.4380.1*.

## MATERIALS AND METHODS

### Identification of lincRNAs.

Our most recent genome assembly and annotation of *R. cerealis* were used (24). Published dual-time-course RNA-Seq data were reanalyzed to identify lincRNAs involved in *R. cerealis* infection and growth (24). These data comprised 18 samples. Wheat leaves inoculated with *R. cerealis* were harvested at 7, 14, and 21 dpi, with three biological replicates for the treatment. Hyphae grown on potato dextrose agar (PDA) medium were collected simultaneously as the control. All raw data containing adaptors and low-quality reads were removed by using Fastp v0.20.1 (48). Next, the clean reads were aligned to the reference genome Rce_V1 using HISAT2 v2.2.1 (49). The alignments were assembled using StringTie v2.1.4 (50) with the available *R. cerealis* annotation data as a reference. Next, the assembled transcripts were merged into a uniform set of transcripts for all samples by using StringTie. Subsequently, Gffcompare v0.10.1 (51) was used to annotate the assembled transcripts, and transcripts with class code “u” were selected. Furthermore, transcripts with lengths of <200 nt were excluded. The coding potentials for the remaining transcripts were then predicted by using CPC2 (Coding Potential Calculator 2) (52), CNCI (Coding-Noncoding Index) (53), and Pfamscan (E value of <1e−5). Transcripts with no coding potential shared by the three software were retained. Finally, the remaining transcripts with a particular expression level (FPKM of >1 in at least one RNA-Seq library) were retained as candidate lincRNAs.

### Analysis of differential expression patterns.

The count matrix of lincRNAs was obtained using the prepDE.py program in StringTie. Differentially expressed lincRNAs were screened with the criteria of an adjusted *P* value of <0.05 and a |log_2_ fold change| of ≥1 using the DESeq2 R package (54).

### Weighted correlation network analysis.

The expression data for DEGs derived from a previous study by Zeng et al. (24) and DELs were extracted to construct a coexpression network using the WGCNA (weighted correlation network analysis) R package (55) with a soft-threshold power of 13 and a minimum module size of 30. Other parameters were set to default values.

### Functional enrichment analysis.

The GO enrichment and KEGG pathway analyses were performed by using the clusterProfiler R package (56). The GO terms and KEGG pathways with *P* values of <0.05 were significantly enriched.

### Identification of hub lincRNAs.

For the blue module, we calculated the connectivity of each gene based on its intramodular connectivity. lincRNAs with an intramodular connectivity value of >0.4 and a degree of >50 were defined as hub lincRNAs. Cytoscape v3.8.2 was used for network visualization (57).

### RNA preparation and expression analysis.

To define the temporal expression patterns of the lincRNAs during wheat-*R. cerealis* interactions, inoculated leaves were harvested at 2, 4, 6, 8, 10, 12, and 14 dpi. As a control, the hyphae of *R. cerealis in vitro* (PDA) were collected at the time of inoculation (0 dpi). Specific qRT-PCR primers (see Table S1 in the supplemental material) were designed to distinguish each lincRNA.

A spin column fungal total RNA purification kit (Sangon Biotech, China) was used to extract RNA. qRT-PCR was performed on the CFX Connect real-time system (Bio-Rad, USA) according to a procedure described previously by Yang et al. (58). The relative expression levels of target lincRNAs in *R. cerealis* were determined using the 2^−ΔΔ^*^CT^* method (59); the expression levels of all of these lincRNAs were normalized to the expression level of *RcActin*. Each sample was analyzed in three biological replicates.

### Potential target gene prediction.

Based on the genome location of *MSTRG.4380.1* relative to the neighboring genes, the protein-coding genes transcribed within a 10-kb window upstream and a 20-kb window downstream were screened as potential *cis*-regulated target genes with the window function of BEDTools software (60).

### BSMV-HIGS assays.

The wheat variety Fielder was selected for BSMV-induced gene silencing in the target lincRNAs. A total of 30 plots of wheat seedlings were used in the experiment, with 12 seedlings in each plot. Among these 30 plots of wheat seedlings, 6, 6, 6, 6, 4, and 2 plots were used for *linc6*, *linc7*, *lic9*, γ, mock, and *TaPDS*, respectively.

The capping and *in vitro* transcription of seven BSMV plasmids (BSMV::α, BSMV::β, BSMV::γ, BSMV::*linc6*, BSMV::*linc7*, BSMV::*linc9*, and BSMV::*TaPDS*) were performed by using the Ribom7G cap analog and the RiboMAX large-scale RNA production system T7 (Promega, USA). The linearized *in vitro* transcripts of five fragments (BSMV::γ, BSMV::*linc6*, BSMV::*linc7*, BSMV::*linc9*, and BSMV::*TaPDS*) were individually mixed with BSMV:α and BSMV::β and then inoculated onto the second expanded leaves of the wheat seedlings (58). These virus-infected plants were maintained in a temperature-controlled chamber at 25°C.

After 14 days, the fourth leaves of wheat with mild chlorotic mosaic symptoms were inoculated with *R. cerealis* isolate R0301 mycelium plugs and maintained at 23°C as described previously by Li et al. (41). The infected fourth leaves were harvested at 3 and 5 dpi for the qRT-PCR study. The fungal biomass was determined by qRT-PCR, as previously described by Huai et al. (61). After 7 days of inoculation, the phenotypes of the inoculated leaves were photographed.

### RNA and sRNA library construction and sequencing.

The infected fourth leaves treated with BSMV:*linc9* and BSMV:γ were harvested at 5 dpi for RNA and sRNA sequencing (three repeats for each treatment). The construction and sequencing of RNA and unique molecular identifiers small RNA were carried out by BGI (http://www.bgitechsolutions.com/).

### RNA-Seq analysis.

After sequencing, the clean reads were retained after the removal of adaptor sequences, contamination, and low-quality reads from raw reads by using SOAPnuke (62). Next, the quality of the data was examined by using fastQC. The clean reads were aligned to the *R. cerealis* reference genome using HISAT2 (49), and read counts and FPKM were determined by using featureCounts for gene expression. Significant differential expression was defined as a |log2 fold change| of ≥1 and a *P*_adj_ value of ≤0.05 using DESeq2. GO categories matched with significantly up- and downregulated genes were identified as described above.

### Identification of miRNAs and target prediction of miRNAs.

After sequencing, the raw reads were filtered by using SOAPnuke (62). Data filtering includes the removal of adaptor sequences, contamination, and low-quality reads from raw reads. Next, clean reads were annotated by using the cmscan program of the Infernal package (63, 64), and reads with high similarities with rRNAs, tRNAs, snRNAs, and snoRNAs were removed. miRDeep2 was used to identify novel miRNAs with no related species in miRbase and quantify miRNAs (65). The potential targets of miRNAs were predicted by using psRobot with default parameters (26).

### Data availability.

Raw sequencing data are available at the National Genomics Data Center (NGDC) (https://ngdc.cncb.ac.cn/) under BioProject accession number PRJCA014565.

## References

[1] Li W, Sun H, Deng Y, Zhang A, Chen H. 2014. The heterogeneity of the rDNA-ITS sequence and its phylogeny in *Rhizoctonia cerealis*, the cause of sharp eyespot in wheat. Curr Genet 60:1–9. doi:10.1007/s00294-013-0397-7. 23839120

[2] Burpee LL, Sanders PL, Cole H, Sherwood RT. 1980. Anastomosis groups among isolates of *Ceratobasidium cornigerum* and related fungi. Mycologia 72:689–701. doi:10.1080/00275514.1980.12021238.

[3] Tomaso-Peterson M, Trevathan LE. 2007. Characterization of *Rhizoctonia*-like fungi isolated from agronomic crops and turfgrasses in Mississippi. Plant Dis 91:260–265. doi:10.1094/PDIS-91-3-0260. 30780558

[4] Chen L, Zhang Z, Liang H, Liu H, Du L, Xu H, Xin Z. 2008. Overexpression of *TiERF1* enhances resistance to sharp eyespot in transgenic wheat. J Exp Bot 59:4195–4204. doi:10.1093/jxb/ern259. 18953072 PMC2639029

[5] Hamada MS, Yin Y, Chen H, Ma Z. 2011. The escalating threat of *Rhizoctonia cerealis*, the causal agent of sharp eyespot in wheat. Pest Manag Sci 67:1411–1419. doi:10.1002/ps.2236. 21726039

[6] Zhu X, Lu C, Du L, Ye X, Liu X, Coules A, Zhang Z. 2017. The wheat NB-LRR gene *TaRCR1* is required for host defence response to the necrotrophic fungal pathogen *Rhizoctonia cerealis*. Plant Biotechnol J 15:674–687. doi:10.1111/pbi.12665. 27862842 PMC5425395

[7] Moya-Elizondo E, Arismendi N, Castro MP, Doussoulin H. 2015. Distribution and prevalence of crown rot pathogens affecting wheat crops in southern Chile. Chil J Agric Res 75:78–84. doi:10.4067/S0718-58392015000100011.

[8] Sun H-Y, Lu C-Q, Li W, Deng Y-Y, Chen H-G. 2017. Homozygous and heterozygous point mutations in succinate dehydrogenase subunits b, c and d of *Rhizoctonia cerealis* conferring resistance to thifluzamide. Pest Manag Sci 73:896–903. doi:10.1002/ps.4361. 27415408

[9] Rinn JL, Chang HY. 2012. Genome regulation by long noncoding RNAs. Annu Rev Biochem 81:145–166. doi:10.1146/annurev-biochem-051410-092902. 22663078 PMC3858397

[10] Kung JTY, Colognori D, Lee JT. 2013. Long noncoding RNAs: past, present, and future. Genetics 193:651–669. doi:10.1534/genetics.112.146704. 23463798 PMC3583990

[11] Mercer TR, Mattick JS. 2013. Structure and function of long noncoding RNAs in epigenetic regulation. Nat Struct Mol Biol 20:300–307. doi:10.1038/nsmb.2480. 23463315

[12] Nejat N, Mantri N. 2018. Emerging roles of long noncoding RNAs in plant response to biotic and abiotic stresses. Crit Rev Biotechnol 38:93–105. doi:10.1080/07388551.2017.1312270. 28423944

[13] Tang J, Chen X, Yan Y, Huang J, Luo C, Tom H, Zheng L. 2021. Comprehensive transcriptome profiling reveals abundant long non-coding RNAs associated with development of the rice false smut fungus, *Ustilaginoidea virens*. Environ Microbiol 23:4998–5013. doi:10.1111/1462-2920.15432. 33587785

[14] Mattick JS, Rinn JL. 2015. Discovery and annotation of long noncoding RNAs. Nat Struct Mol Biol 22:5–7. doi:10.1038/nsmb.2942. 25565026

[15] Jiang N, Cui J, Shi Y, Yang G, Zhou X, Hou X, Meng J, Luan Y. 2019. Tomato *lncRNA23468* functions as a competing endogenous RNA to modulate *NBS-LRR* genes by decoying *miR482b* in the tomato-*Phytophthora infestans* interaction. Hortic Res 6:28. doi:10.1038/s41438-018-0096-0. 30729018 PMC6355781

[16] Cui J, Luan Y, Jiang N, Bao H, Meng J. 2017. Comparative transcriptome analysis between resistant and susceptible tomato allows the identification of *lncRNA16397* conferring resistance to *Phytophthora infestans* by co-expressing glutaredoxin. Plant J 89:577–589. doi:10.1111/tpj.13408. 27801966

[17] Zhang B, Su T, Li P, Xin X, Cao Y, Wang W, Zhao X, Zhang D, Yu Y, Li D, Yu S, Zhang F. 2021. Identification of long noncoding RNAs involved in resistance to downy mildew in Chinese cabbage. Hortic Res 8:44. doi:10.1038/s41438-021-00479-1. 33642586 PMC7917106

[18] Seo JS, Sun H-X, Park BS, Huang C-H, Yeh S-D, Jung C, Chua N-H. 2017. ELF18-INDUCED LONG-NONCODING RNA associates with mediator to enhance expression of innate immune response genes in Arabidopsis. Plant Cell 29:1024–1038. doi:10.1105/tpc.16.00886. 28400491 PMC5466027

[19] Seo JS, Diloknawarit P, Park BS, Chua N-H. 2019. ELF18-iNDUCED LONG NONCODING RNA 1 evicts fibrillarin from mediator subunit to enhance PATHOGENESIS-RELATED GENE 1 (PR1) expression. New Phytol 221:2067–2079. doi:10.1111/nph.15530. 30307032

[20] Ai G, Li T, Zhu H, Dong X, Fu X, Xia C, Pan W, Jing M, Shen D, Xia A, Tyler BM, Dou D. 2023. *BPL3* binds the long noncoding RNA *nalncFL7* to suppress *FORKED-LIKE7* and modulate HAI1-mediated MPK3/6 dephosphorylation in plant immunity. Plant Cell 35:598–616. doi:10.1093/plcell/koac311. 36269178 PMC9806616

[21] Li R, Xue H-S, Zhang D-D, Wang D, Song J, Subbarao KV, Klosterman SJ, Chen J-Y, Dai X-F. 2022. Identification of long non-coding RNAs in *Verticillium dahliae* following inoculation of cotton. Microbiol Res 257:126962. doi:10.1016/j.micres.2022.126962. 35042052

[22] Till P, Mach RL, Mach-Aigner AR. 2018. A current view on long noncoding RNAs in yeast and filamentous fungi. Appl Microbiol Biotechnol 102:7319–7331. doi:10.1007/s00253-018-9187-y. 29974182 PMC6097775

[23] Li J, Liu X, Yin Z, Hu Z, Zhang K-Q. 2021. An overview on identification and regulatory mechanisms of long non-coding RNAs in fungi. Front Microbiol 12:638617. doi:10.3389/fmicb.2021.638617. 33995298 PMC8113380

[24] Zeng Q, Cao W, Li W, Wu J, Figueroa M, Liu H, Qin G, Wang Q, Yang L, Zhou Y, Yu Y, Huang L, Liu S, Luo Y, Mu Z, Li X, Liu J, Wang X, Wang C, Yuan F, Chen H, Xu H, Dodds PN, Han D, Kang Z. 2022. Near telomere-to-telomere nuclear phased chromosomes of the dikaryotic wheat fungus *Rhizoctonia cerealis*. bioRxiv. doi:10.1101/2022.03.18.484966.

[25] Sen R, Ghosal S, Das S, Balti S, Chakrabarti J. 2014. Competing endogenous RNA: the key to posttranscriptional regulation. ScientificWorldJournal 2014:896206. doi:10.1155/2014/896206. 24672386 PMC3929601

[26] Wu H-J, Ma Y-K, Chen T, Wang M, Wang X-J. 2012. PsRobot: a Web-based plant small RNA meta-analysis toolbox. Nucleic Acids Res 40:W22–W28. doi:10.1093/nar/gks554. 22693224 PMC3394341

[27] Donaldson ME, Saville BJ. 2013. Ustilago maydis natural antisense transcript expression alters mRNA stability and pathogenesis. Mol Microbiol 89:29–51. doi:10.1111/mmi.12254. 23650872 PMC3739942

[28] Wang J, Zeng W, Xie J, Fu Y, Jiang D, Lin Y, Chen W, Cheng J. 2021. A novel antisense long non-coding RNA participates in asexual and sexual reproduction by regulating the expression of *GzmetE* in *Fusarium graminearum*. Environ Microbiol 23:4939–4955. doi:10.1111/1462-2920.15399. 33438341

[29] Choi G, Jeon J, Lee H, Zhou S, Lee Y-H. 2022. Genome-wide profiling of long non-coding RNA of the rice blast fungus *Magnaporthe oryzae* during infection. BMC Genomics 23:132. doi:10.1186/s12864-022-08380-4. 35168559 PMC8845233

[30] Jain P, Sharma V, Dubey H, Singh PK, Kapoor R, Kumari M, Singh J, Pawar DV, Bisht D, Solanke AU, Mondal TK, Sharma TR. 2017. Identification of long non-coding RNA in rice lines resistant to rice blast pathogen *Maganaporthe* [*sic*] *oryzae*. Bioinformation 13:249–255. doi:10.6026/97320630013249. 28959093 PMC5609289

[31] Luo S, Lu JY, Liu L, Yin Y, Chen C, Han X, Wu B, Xu R, Liu W, Yan P, Shao W, Lu Z, Li H, Na J, Tang F, Wang J, Zhang YE, Shen X. 2016. Divergent lncRNAs regulate gene expression and lineage differentiation in pluripotent cells. Cell Stem Cell 18:637–652. doi:10.1016/j.stem.2016.01.024. 26996597

[32] Cemel IA, Ha N, Schermann G, Yonekawa S, Brunner M. 2017. The coding and noncoding transcriptome of *Neurospora crassa*. BMC Genomics 18:978. doi:10.1186/s12864-017-4360-8. 29258423 PMC5738166

[33] Liu N, Wang P, Li X, Pei Y, Sun Y, Ma X, Ge X, Zhu Y, Li F, Hou Y. 2022. Long non-coding RNAs profiling in pathogenesis of *Verticillium dahliae*: new insights in the host-pathogen interaction. Plant Sci 314:111098. doi:10.1016/j.plantsci.2021.111098. 34895536

[34] Wang Y, Ye W, Wang Y. 2018. Genome-wide identification of long non-coding RNAs suggests a potential association with effector gene transcription in *Phytophthora sojae*. Mol Plant Pathol 19:2177–2186. doi:10.1111/mpp.12692. 29665235 PMC6638102

[35] Nowara D, Gay A, Lacomme C, Shaw J, Ridout C, Douchkov D, Hensel G, Kumlehn J, Schweizer P. 2010. HIGS: host-induced gene silencing in the obligate biotrophic fungal pathogen Blumeria graminis. Plant Cell 22:3130–3141. doi:10.1105/tpc.110.077040. 20884801 PMC2965548

[36] Panwar V, McCallum B, Bakkeren G. 2013. Host-induced gene silencing of wheat leaf rust fungus *Puccinia triticina* pathogenicity genes mediated by the barley stripe mosaic virus. Plant Mol Biol 81:595–608. doi:10.1007/s11103-013-0022-7. 23417582

[37] Koch A, Kumar N, Weber L, Keller H, Imani J, Kogel KH. 2013. Host-induced gene silencing of cytochrome P450 lanosterol C14alpha-demethylase-encoding genes confers strong resistance to Fusarium species. Proc Natl Acad Sci USA 110:19324–19329. doi:10.1073/pnas.1306373110. 24218613 PMC3845197

[38] Yin C, Jurgenson JE, Hulbert SH. 2011. Development of a host-induced RNAi system in the wheat stripe rust fungus *Puccinia striiformis* f. sp. *tritici*. Mol Plant Microbe Interact 24:554–561. doi:10.1094/MPMI-10-10-0229. 21190437

[39] Xu J, Wang X, Li Y, Zeng J, Wang G, Deng C, Guo W. 2018. Host-induced gene silencing of a regulator of G protein signalling gene (*VdRGS1*) confers resistance to *Verticillium* wilt in cotton. Plant Biotechnol J 16:1629–1643. doi:10.1111/pbi.12900. 29431919 PMC6096726

[40] Hu D, Chen Z-Y, Zhang C, Ganiger M. 2020. Reduction of Phakopsora pachyrhizi infection on soybean through host- and spray-induced gene silencing. Mol Plant Pathol 21:794–807. doi:10.1111/mpp.12931. 32196911 PMC7214474

[41] Li X, Mu K, Yang S, Wei J, Wang C, Yan W, Yuan F, Wang H, Han D, Kang Z, Zeng Q. 2022. Reduction of Rhizoctonia cerealis infection on wheat through host- and spray-induced gene silencing of an orphan secreted gene. Mol Plant Microbe Interact 35:803–813. doi:10.1094/MPMI-04-22-0075-R. 36102883

[42] Cai Q, He B, Wang S, Fletcher S, Niu D, Mitter N, Birch PRJ, Jin H. 2021. Message in a bubble: shuttling small RNAs and proteins between cells and interacting organisms using extracellular vesicles. Annu Rev Plant Biol 72:497–524. doi:10.1146/annurev-arplant-081720-010616. 34143650 PMC8369896

[43] Qiao L, Lan C, Capriotti L, Ah-Fong A, Nino Sanchez J, Hamby R, Heller J, Zhao H, Glass NL, Judelson HS, Mezzetti B, Niu D, Jin H. 2021. Spray-induced gene silencing for disease control is dependent on the efficiency of pathogen RNA uptake. Plant Biotechnol J 19:1756–1768. doi:10.1111/pbi.13589. 33774895 PMC8428832

[44] Wang M, Thomas N, Jin H. 2017. Cross-kingdom RNA trafficking and environmental RNAi for powerful innovative pre- and post-harvest plant protection. Curr Opin Plant Biol 38:133–141. doi:10.1016/j.pbi.2017.05.003. 28570950 PMC5720367

[45] Yang L, Froberg JE, Lee JT. 2014. Long noncoding RNAs: fresh perspectives into the RNA world. Trends Biochem Sci 39:35–43. doi:10.1016/j.tibs.2013.10.002. 24290031 PMC3904784

[46] Liu J, Wang H, Chua N-H. 2015. Long noncoding RNA transcriptome of plants. Plant Biotechnol J 13:319–328. doi:10.1111/pbi.12336. 25615265

[47] Zhang Y-C, Liao J-Y, Li Z-Y, Yu Y, Zhang J-P, Li Q-F, Qu L-H, Shu W-S, Chen Y-Q. 2014. Genome-wide screening and functional analysis identify a large number of long noncoding RNAs involved in the sexual reproduction of rice. Genome Biol 15:512. doi:10.1186/s13059-014-0512-1. 25517485 PMC4253996

[48] Chen S, Zhou Y, Chen Y, Gu J. 2018. Fastp: an ultra-fast all-in-one FASTQ preprocessor. Bioinformatics 34:i884–i890. doi:10.1093/bioinformatics/bty560. 30423086 PMC6129281

[49] Kim D, Paggi JM, Park C, Bennett C, Salzberg SL. 2019. Graph-based genome alignment and genotyping with HISAT2 and HISAT-genotype. Nat Biotechnol 37:907–915. doi:10.1038/s41587-019-0201-4. 31375807 PMC7605509

[50] Kovaka S, Zimin AV, Pertea GM, Razaghi R, Salzberg SL, Pertea M. 2019. Transcriptome assembly from long-read RNA-seq alignments with StringTie2. Genome Biol 20:278. doi:10.1186/s13059-019-1910-1. 31842956 PMC6912988

[51] Pertea G, Pertea M. 2020. GFF utilities: GffRead and GffCompare. F1000Res 9:304. doi:10.12688/f1000research.23297.2. PMC7222033 32489650

[52] Kang Y-J, Yang D-C, Kong L, Hou M, Meng Y-Q, Wei L, Gao G. 2017. CPC2: a fast and accurate coding potential calculator based on sequence intrinsic features. Nucleic Acids Res 45:W12–W16. doi:10.1093/nar/gkx428. 28521017 PMC5793834

[53] Sun L, Luo H, Bu D, Zhao G, Yu K, Zhang C, Liu Y, Chen R, Zhao Y. 2013. Utilizing sequence intrinsic composition to classify protein-coding and long non-coding transcripts. Nucleic Acids Res 41:e166. doi:10.1093/nar/gkt646. 23892401 PMC3783192

[54] Love MI, Huber W, Anders S. 2014. Moderated estimation of fold change and dispersion for RNA-seq data with DESeq2. Genome Biol 15:550. doi:10.1186/s13059-014-0550-8. 25516281 PMC4302049

[55] Langfelder P, Horvath S. 2008. WGCNA: an R package for weighted correlation network analysis. BMC Bioinformatics 9:559. doi:10.1186/1471-2105-9-559. 19114008 PMC2631488

[56] Yu G, Wang L-G, Han Y, He Q-Y. 2012. ClusterProfiler: an R package for comparing biological themes among gene clusters. OMICS 16:284–287. doi:10.1089/omi.2011.0118. 22455463 PMC3339379

[57] Shannon P, Markiel A, Ozier O, Baliga NS, Wang JT, Ramage D, Amin N, Schwikowski B, Ideker T. 2003. Cytoscape: a software environment for integrated models of biomolecular interaction networks. Genome Res 13:2498–2504. doi:10.1101/gr.1239303. 14597658 PMC403769

[58] Yang Q, Huai B, Lu Y, Cai K, Guo J, Zhu X, Kang Z, Guo J. 2020. A stripe rust effector *Pst18363* targets and stabilises *TaNUDX23* that promotes stripe rust disease. New Phytol 225:880–895. doi:10.1111/nph.16199. 31529497

[59] Livak KJ, Schmittgen TD. 2001. Analysis of relative gene expression data using real-time quantitative PCR and the 2(−Delta Delta C(T)) method. Methods 25:402–408. doi:10.1006/meth.2001.1262. 11846609

[60] Quinlan AR, Hall IM. 2010. BEDTools: a flexible suite of utilities for comparing genomic features. Bioinformatics 26:841–842. doi:10.1093/bioinformatics/btq033. 20110278 PMC2832824

[61] Huai B, Yang Q, Qian Y, Qian W, Kang Z, Liu J. 2019. ABA-induced sugar transporter *TaSTP6* promotes wheat susceptibility to stripe rust. Plant Physiol 181:1328–1343. doi:10.1104/pp.19.00632. 31540949 PMC6836835

[62] Chen Y, Chen Y, Shi C, Huang Z, Zhang Y, Li S, Li Y, Ye J, Yu C, Li Z, Zhang X, Wang J, Yang H, Fang L, Chen Q. 2018. SOAPnuke: a MapReduce acceleration-supported software for integrated quality control and preprocessing of high-throughput sequencing data. Gigascience 7:gix120. doi:10.1093/gigascience/gix120. 29220494 PMC5788068

[63] Nawrocki EP, Kolbe DL, Eddy SR. 2009. Infernal 1.0: inference of RNA alignments. Bioinformatics 25:1335–1337. doi:10.1093/bioinformatics/btp157. 19307242 PMC2732312

[64] Nawrocki EP, Eddy SR. 2013. Infernal 1.1: 100-fold faster RNA homology searches. Bioinformatics 29:2933–2935. doi:10.1093/bioinformatics/btt509. 24008419 PMC3810854

[65] Friedlander MR, Mackowiak SD, Li N, Chen W, Rajewsky N. 2012. miRDeep2 accurately identifies known and hundreds of novel microRNA genes in seven animal clades. Nucleic Acids Res 40:37–52. doi:10.1093/nar/gkr688. 21911355 PMC3245920

