# Supplementary material for: Identification of Long Intergenic Noncoding RNAs in Rhizoctonia cerealis following Inoculation of Wheat
Source: Microbiol Spectr. 2023 Apr 10;11(3):e03449-22. doi: 10.1128/spectrum.03449-22 (PMC10269763; doi:10.1128/spectrum.03449-22)
Supplement: Supplemental file 5 — Supplemental material. Download spectrum.03449-22-s0005.pdf, PDF file, 0.2 MB [file spectrum.03449-22-s0005.pdf]

Supporting information legends

Table S1 Primers used in this study

Fig.S1 Intersection of non-coding RNAs identified by three different software, CNCI, CPC2 and PFAM

Fig.S2 GO enrichment analysis of 111 upregulated genes

Fig.S3 Small RNA length distribution. The X-axis is the length of small RNAs, and the Y-axis is the corresponding number of small RNAs. The length distribution can reflect the types of small RNAs in the sequencing data.

DataSet S1 1319 R. cerealis lincRNAs identified in this study

DataSet S2 The annotation of genes enriched 11 modules produced by WGCNA analysis

DataSet S3 Genes closed to *MSTRG.4380.1* and co-expressed with *MSTRG.4380.1*

DataSet S4 23 novel miRNAs identified in this study

Table S1 Primers in this study

Primer name	Sequence (5'→3')	Purpose
RcLINC1-qRT-F	CCCTTGTGAAACCCATGGAT	qRT-PCR
RcLINC1-qRT-R	TCTCGGACTGTTCTCGGACC	
RcLINC2-qRT-F	GATATCCAAAGTCTAACATG	
RcLINC2-qRT-R	GCTCGGGGAAACAACAAGTG	
RcLINC3-qRT-F	CGGAAAAGAGAGTGATAAGG	
RcLINC3-qRT-R	ATCTACAAACATGGCAGAAA	
RcLINC4-qRT-F	TGCGTGGTTTAGGTTTTAGA	
RcLINC4-qRT-R	ACGAGCGATTGGTTATTGTC	
RcLINC5-qRT-F	TCTACACTTGCTCCATCGTC	
RcLINC5-qRT-R	CTTGTCTTCCGCCTTGACTT	
RcLINC6-qRT-F	GTCGCTCCGCCGCCTAACAC	
RcLINC6-qRT-R	ACAAGAATATCGCCACATCA	
RcLINC7-qRT-F	CCCTCTGACTTCTCTCTCGC	
RcLINC7-qRT-R	TGTTCTCCCTCTCATGCTCC	
RcLINC8-qRT-F	ACTACGACCCCACCTTGTCT	
RcLINC8-qRT-R	AACACACCAGCACACTCGAA	
RcLINC9-qRT-F	GCTGTTCCCGTGATCAATGC	
RcLINC9-qRT-R	TGTTTCGCATGCACTATGGAC	
y-RcLINC6-F	tagctagctgattaattaaTGGTGTGACAGTCCAAAGTCCA	HIGS (host-induced gene silencing)
y-RcLINC6-R	ttgctagctgagcggccgcCAACAACGTGCTCACCCAGC	
y-RcLINC7-F	tagctagctgattaattaaTGGTGCCCCCTCTGACTTC	
y-RcLINC7-R	ttgctagctgagcggccgcGGATTTTTGATTTTTATCAAACTTAGTTG	
y-RcLINC9-F	tagctagctgattaattaaTGTTGGCATTCGCCGTATCA	
y-RcLINC9-R	ttgctagctgagcggccgcAGGCTTCATTTGCTTCGATCC	

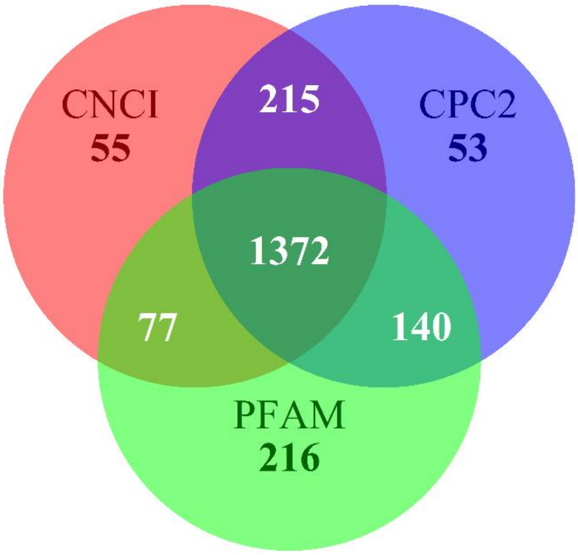

Fig.S1 Intersection of non-coding RNAs identified by three different software, CNCI, CPC2 and PFAM.

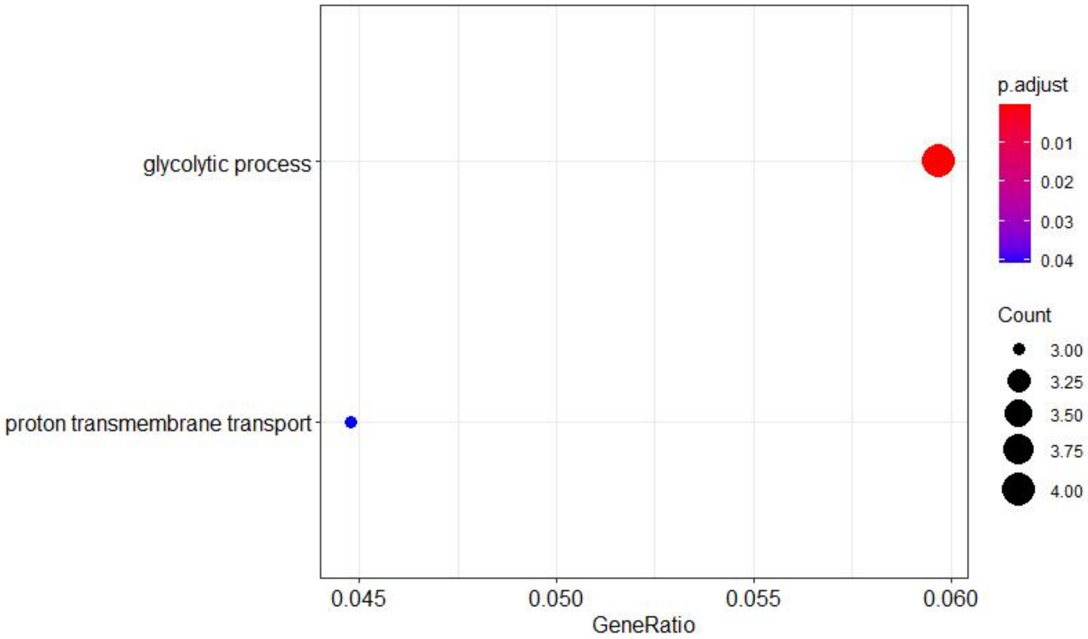

Fig.S2 GO enrichment analysis of 111 upregulated genes.

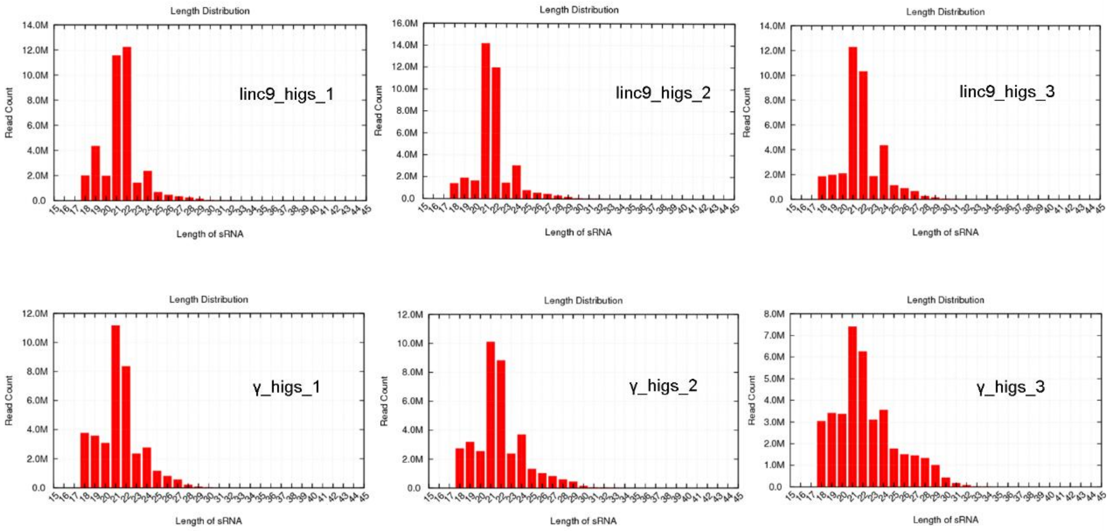

Fig.S3 Small RNA length distribution.
